# “ASUKI Step” pedometer intervention in university staff: rationale and design

**DOI:** 10.1186/1471-2458-12-657

**Published:** 2012-08-15

**Authors:** Barbara E Ainsworth, Cheryl Der Ananian, Ali Soroush, Jenelle Walker, Pamela Swan, Eric Poortvliet, Agneta Yngve

**Affiliations:** 1 Exercise and Wellness Program, School of Nutrition and Health Promotion, Arizona State University, 500 N. 3rd Street, Phoenix, AZ 85003, USA; 2 Department of Biosciences and Nutrition, Unit for Public Health Nutrition, Karolinska Institutet, Stockholm, Sweden; 3 Kermanshah University of Medical Sciences (KUMS), Kermanshah, Iran; 4 Oslo and Akershus University College of Applied Sciences, Postbox 4, St. Olavs plass, NO-0130, Oslo, Norway

**Keywords:** Physical activity, Exercise, Worksite health promotion, Physical fitness

## Abstract

**Background:**

We describe the study design and methods used in a 9-month pedometer-based worksite intervention called “ASUKI Step” conducted at the Karolinska Institutet (KI) in Stockholm, Sweden and Arizona State University (ASU) in the greater Phoenix area, Arizona.

**Methods/design:**

“ASUKI Step” was based on the theory of social support and a quasi-experimental design was used for evaluation. Participants included 2,118 faculty, staff, and graduate students from ASU (n = 712) and KI (n = 1,406) who participated in teams of 3–4 persons. The intervention required participants to accumulate 10,000 steps each day for six months, with a 3-month follow-up period. Steps were recorded onto a study-specific website. Participants completed a website-delivered questionnaire four times to identify socio-demographic, health, psychosocial and environmental correlates of study participation. One person from each team at each university location was randomly selected to complete physical fitness testing to determine their anthropometric and cardiovascular health and to wear an accelerometer for one week. Study aims were: 1) to have a minimum of 400 employee participants from each university site reach a level of 10, 000 steps per day on at least 100 days (3.5 months) during the trial period; 2) to have 70% of the employee participants from each university site maintain two or fewer inactive days per week, defined as a level of less than 3,000 steps per day; 3) to describe the socio-demographic, psychosocial, environmental and health-related determinants of success in the intervention; and 4) to evaluate the effects of a pedometer-based walking intervention in a university setting on changes in self-perceived health and stress level, sleep patterns, anthropometric measures and fitness.

Incentives were given for compliance to the study protocol that included weekly raffles for participation prizes and a grand finale trip to Arizona or Sweden for teams with most days over 10,000 steps.

**Discussion:**

“ASUKI Step” is designed to increase the number of days employees walk 10,000 steps and to reduce the number of days employees spend being inactive. The study also evaluates the intra- and interpersonal determinants for success in the intervention and in a sub-sample of the study, changes in physical fitness and body composition during the study.

**Trial registration:**

Current Controlled Trials NCT01537939

## Background

Physical inactivity is recognized as a major public health problem [1] as it is a risk factor for chronic disease and is responsible for 2 million early deaths per year worldwide [2]. Between 70% and 90% of early death from the major killers in the US; coronary heart disease, colon cancer and type 2-diabetes [3] are believed to be caused by poor nutrition, sedentary living, and tobacco use and are considered preventable [4]. These lifestyle factors appear to play a prominent role in the mechanisms and processes that lead to the development of many chronic diseases. The largest reductions in chronic disease prevalence will be achieved when individuals adopt and maintain lifestyles that include a healthy diet and regular physical activity [5].

Physical activity is defined as “any body movement produced by skeletal muscle that results in a substantial increase over the resting energy expenditure” [6]. Lifestyle modification that includes regular physical activity is the foundation of prevention and treatment of cardiovascular disease and diabetes. A moderate level of physical activity decreases the risk of diabetes and cardiovascular disease, compared with individuals of the same weight but with a lower level of physical activity [7,8]. Several behavior modification programs targeting diet and physical activity have been developed [9-12]. Essential elements of these programs typically include problem solving, goal formulation, extended behavioral control, and social support combined with a lifestyle intervention. Programs targeting both dietary and physical activity habits have the most pronounced effects, both initially and after one year [9].

Worksites are ideal settings to promote physical activity. Worksites often provide opportunities for physical activity through on-site facilities, trails or locations for exercise and may also provide social support for employees to engage in physical activity. Employee wellness programs are effective in helping to control health care costs associated with illness, disability, and disease. Accordingly, most employers recognize that employer-sponsored health promotion activities are an effective way to reduce health care cost due to modifiable risk factors and to increase productivity [13].

Worksite-based employee health promotion programs are recommended by the U.S. Guide for Community Preventive Services to improve physical activity, reduce obesity and prevent chronic disease in adults [14]. Recommended worksite approaches focusing on physical activity include the creation of or enhanced access to places for physical activity combined with informational outreach activities, worksite programs to control overweight and obesity, and point of decision prompts to encourage the use of stairs. Informational and educational strategies that increase knowledge about healthy diets and physical activity, behavioral and social techniques that target the cognitions (e.g. awareness, self-efficacy) and social factors that influence behavior change, as well as policy and environmental approaches that make healthy choices easier for the entire workforce by changing physical or organizational structures are all potential methods that can be utilized in worksite wellness programs.

Office-based employees are relatively inactive and are estimated to accumulate 4,000-6,000 steps per day [15]. This is in contrast to 8,000 – 10,000 steps per day recommended for healthy adults [16]. Pedometer-based interventions have been used to increase walking and promote physical activity behavior changes in worksite settings with mixed effectiveness [17-21]. One pedometer-based intervention conducted in a university setting showed a statistically significant increase in the mean number of steps walked per day, a significant reduction in mean BMI and a 3.4% reduction in the prevalence of hypertension in university staff [19]. Likewise, significant increases in steps per day were obtained in university employees randomized to either accumulate steps “in tasks” throughout the day or by taking 15 minute walks on designated walking loops [17]. However, in the latter study no significant improvements were obtained for blood pressure, body fat percent or waist circumference. In a 20-week pedometer-based physical activity program that was built on the Social Ecological Model [20], a decrease in the number of steps taken from baseline to the end of the intervention was observed and the authors of this study postulated that the observed decline in step counts was attributable to seasonality as the baseline step counts were recorded in late summer and post-test steps were recorded in winter. Additionally, participants who were active at baseline (> 10,000 steps per day) at the intervention worksite saw a smaller decline in steps taken per day compared to active participants at the comparison worksite. Speck and colleagues [21] examined the feasibility of a pedometer-based 10,000 steps per day intervention at a Midwestern University and demonstrated relatively low completion (26%) and adherence (9%) rates. A recent systematic review of interventions designed to promote walking suggests that the most successful walking interventions are those that are tailored to the individual’s needs, targeted to individuals who are the most sedentary or the most motivated to change and delivered at the individual level or through groups [22].

We developed and conducted an evidence-based, pedometer driven intervention called “ASUKI Step” that was grounded in social support theory and utilized incentive motivation and goal-setting. The aims of this study were to examine the impact of the program on ambulatory physical activity and physical fitness parameters and to examine the correlates of participation in the study. The aim of this paper was to describe the methods, the design and the theory base of the “ASUKI Step” intervention.

## Methods/design

The overall goal of “ASUKI Step” was to increase physical activity in university employees. “ASUKI Step” was a 9- month study designed to evaluate the impact of a worksite physical activity intervention at increasing physical activity levels, decreasing inactive days and improving physical fitness and health outcomes in university employees. The intervention occurred during the first 24 weeks and was followed by a 12-week follow-up period. The acronym ASUKI represents the collaborative study between Arizona State University (ASU) located in Phoenix, Arizona and the Karolinska Institutet (KI), located in Stockholm, Sweden. The plan for the intervention was to recruit at least 1,400 employees from each university to participate in the program. Participants enrolled in the intervention in teams of three-to-four persons per work unit to enhance social support for physical activity and step counters were the primary intervention tool.

### Study design

The study used a quasi-experimental design and the intervention was based on the theory of social support [23]. Participants were asked to set as a goal to achieve 10,000 steps each day and record accumulated steps onto a study-specific website. During the first, third, sixth and ninth month of the study, participants completed a website-delivered questionnaire to identify socio-demographic, health, psychosocial and environmental correlates of study participation. At the beginning of the study, one person from each team at each university location was randomly selected to complete physical fitness testing. The selected individuals completed successive physical fitness tests and wore the accelerometer for a week at the specified time periods throughout the study. Incentives were given for compliance to the study protocol that included weekly raffles for participation prizes and a grand finale trip to Arizona or Sweden for teams with most days over 10,000 steps.

### Study population and inclusion criteria

The study enrolled 2,118 volunteer faculty, staff, and graduate student employees from ASU (n = 712, 82.5% women) and KI (n = 1,406, 78.0% women). Inclusion criteria were, a) employed at ASU or the KI, b) the ability to read, speak and understand English (ASU only), c) not currently pregnant or lactating, and d) free of physical problems that affect the ability to walk, and e) ages 18 and older. Each university had multiple campuses from which to recruit participants. ASU recruited participants from the Tempe (n = 492), Polytechnic (n = 91), and the Downtown Phoenix campuses (n = 129). KI recruited participants from the Solna (n = 865) and Huddinge campuses (n = 541). The average age for participants was 42.4 ± 12.0 (ASU 41.4 ± 12.0; KI 42.9 ± 12.0). Before starting the study, each person completed a PAR-Q to rule out contraindications for exercise and gave informed consent to engage in a research study. Consent to perform the study was approved by the Institutional Review Boards at ASU and KI.

### Recruitment

Recruitment procedures were similar at both universities. Participants were recruited by announcements placed in university and staff newsletters and through e-mail messages that were sent to faculty, staff, and graduate students employed at the universities. Announcements indicated the purpose, organization, and duration of the study, cost, benefits, the registration website, and where to call for more information about the study. A recruitment meeting was held on each university campus to inform potential participants about the study. A letter of invitation signed by the University leadership (President and Provost at ASU; and the President at KI) was sent to University Deans, Directors, and Department or Program Chairs inviting their faculty and staff to participate in the study. The study details also were posted on the KI website, the KI internal newsletter and the ASU website for selected campuses (Polytechnic and Downtown) and press releases were sent to the local popular newspapers.

### Research design

The study used a pre-post, non-randomized, experimental group only quasi-experimental design.

### Timeline

**Figure 1:**
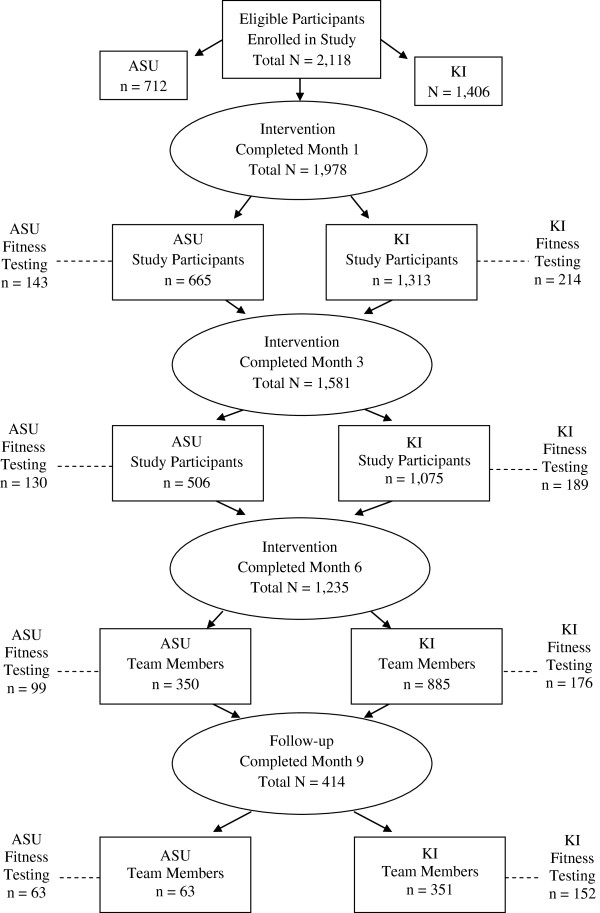
Study flow and participant retention at each level of the study.

“ASUKI Step” was a 6-month pedometer-based intervention lasting from March 16 to September 16, 2009, with a 3 month non-intervention follow-up period (September 16-December 16, 2009). The workplace intervention took place on ASU and KI campuses simultaneously. Questionnaire assessments occurred for all participants at month 1, 3, 6, and 9 and physical fitness assessments occurred for a randomly selected sub-set of participants during the same months. The participant flow from recruitment to completion is presented in Figure 1.

### Theoretical framework

The theoretical construct guiding the walking intervention was social support. According to Cohen et al., social support can be defined as the resources provided by others in different contexts [23]. Social support was fostered by having participants create teams of 3 or 4 persons who worked in the same department or unit to encourage daily walking behaviors and by providing daily feedback of the team’s steps accrued. Goal setting and self-monitoring of physical activity were also encouraged by the intervention as participants were asked to set individual physical activity goals with the ultimate goal of achieving 10,000 steps per day and to monitor and log their daily step counts. Team members could see each others’ steps counts per day and provide support to one another in achieving their step goals. The amount, types and level of social support provided by team members was determined by the individual team members and included such methods as walking with team members, verbal encouragement, with-in team contests and giving each other reminders to be active. Intervention boosters focused on incentive motivation. These boosters included weekly gifts given to teams who recorded their steps walked each day onto the website and a grand prize that was given to the winning team who met the following criteria; a) each team member took at least 10,000 steps per day during the study, and b) had the most steps for the entire team. The grand prize was a trip to Stockholm, Sweden for the ASU winning team and to Phoenix, Arizona for the KI winning team and individuals.

### Outcome measures

Four aims were identified as the main outcomes for the study.

To have a minimum of 400 employee participants from each university site reach a level of 10, 000 steps per day on at least 100 days (3.5 months) during the trial period;

To have 70% of the employee participants from each university site maintain two or fewer inactive days per week, defined as a level of less than 3,000 steps per day;

To describe the socio-demographic, psychosocial, environmental and health-related determinants of success in the intervention;

To evaluate the effects of a pedometer-based walking intervention in a university setting on changes in self-perceived health and stress level, sleep patterns, anthropometric measures and fitness.

### Study protocol

To enroll in the intervention, participants received a registration packet from each university’s study director that contained a pedometer, a website registration number, and instructions how to use the “ASUKI Step” website to enroll in the study and for recording their steps throughout the study. Participants were required to identify two or three co-workers in their department or unit to form a walking team. Each team selected a team name (e.g., “Wonder Walkers”) and individual participants selected a surrogate name (e.g., “Walking Lady”) to identify their step progress on the website. Selecting a surrogate name was encouraged to protect the anonymity of the study participants. At the end of each day, participants were instructed to record their steps on a website developed for the study. In addition to keeping track of all steps recorded, the website provided feedback on steps accrued for each participant and team over time. Individual and team results for each university were posted and constantly updated when new step counts were entered. The individuals and teams with the most steps recorded were placed at the top of the list to serve as a source of motivation for others to maintain participation in the study and to walk more. Posting the participants’ steps accrued during the contest also provided a source of feedback for the study participants to determine their chances for winning the grand prize.

Months 1 to 6 of the study were the intervention period, and months 7 to 9 were the non-intervention follow-up period. At months 1, 3, 6 and 9, participants completed an online questionnaire to assess their self-perceived health status and health behaviors, psychosocial variables, including stress and social support, physical activity patterns, and environmental supports for physical activity. The questionnaire had 101 items and took about 45 minutes to complete.

One person from each team was selected randomly to assess changes in cardiorespiratory fitness, body composition, blood pressure, and accelerometry measured physical activity at months 1, 3, 6, and 9. The purpose of the sub-study was to determine the effects of the intervention on physical fitness and objectively-measured physical activity. Accelerometers were worn for one-week as an objective measure of physical activity that enabled researchers to evaluate the time spent in physical activities by intensity levels. Selected participants were notified by e-mail and invited to a testing laboratory on the ASU or KI campuses where they were employed. The testing session took about 1 hour to complete.

### Study measures and instruments

Accelerometery, pedometer steps, questionnaires, anthropometric, and cardiorespiratory laboratory measures were used to assess the success of “ASUKI Step”. Questionnaire items included descriptive data, perceived health status, social support, self-efficacy for exercise, self reported physical activity levels, report of previous injuries, work place stress, sleep habits, opportunities for exercise at work and home locations, and transportation methods. Pedometer steps and questionnaire responses were recorded on a website developed for the study. The following measures were used.

*ASUKI Step website* was developed and maintained by Select Wellness (Stockholm, Sweden; http://www.selectwellness.com/steg-en/). The website was used to enroll study participants, to complete questionnaire-based assessments, to track steps taken, and to maintain contact with study participants.

*Steps per day* were recorded using a New Lifestyles®, Inc. SW-200 pedometer (Yamax Corporation Tokyo, Japan) that was worn on the participants’ waist band over their right hip every day during the 20-week intervention. Participants were instructed to put the pedometer on in the morning and take it off in the evening or when they were in water. Participation in non-ambulatory activities (e.g., swimming, bicycling) were assigned 2,700 steps per half hour of participation. If a pedometer quit working or was lost, participants paid about $20 for a replacement monitor. The SW-200 pedometer is valid within ± 3% of actual steps taken during a self-paced walk on an individual and within 1% of actual steps for a group mean [24]. This accuracy is similar in normal weight, overweight, and moderately obese adults [25].

#### Questionnaire items

*Demographic data* included 16 questions about self-reported height (cm or ft:in) and weight (kg or lb), age, sex, marital status, education, race/ethnicity (at ASU only), country where parents were born, time spent taking care of others, and postal code. The postal code was obtained as an indicator to compare the participant’s rating of their neighborhood environment for walking.

*Job information* included eight questions developed for this study regarding hours worked per week, job responsibilities, job security, and time spent sitting, standing, and walking at work.

*Health-related quality of life* included an adaption of six questions from the extended core of the EuroQol (EQ-5D) scale which were used to identify self-reported ability to walk unaided, perform self-care and usual activities, and pursue family and leisure activities, and whether the participants experienced physical pain, discomfort, anxiety or depression. They also rated their current health as compared with their health nine months earlier. The EuroQol scale provides a standardized measure of health status in order to provide a simple, generic measure of health for clinical and economic appraisal. The questions were scored by summing the 1–3 point responses (none, moderate, high) and presented a mean score to be used in data analysis. Evaluation of concurrent validity evidence of the EuroQol scale shows correlations on the order of .64 to .71 with five generic health-related quality-of-life scales (Health Utilities Index Mark 2 and Mark 3, Quality of Well-Being Scale Self Administered form, the Health and Activities Limitations index, and the SF-6D based on the SF-36 scale) used in population studies [26].

#### Sleep

Trouble sleeping and the quality of sleep were measured using two questions developed for this study. Participants were asked to rate how frequently they had trouble sleeping using a 5-point Likert scale from never to always, everyday. Likewise, participants were asked to rate their quality of sleep using a 5-point Likert scale from very good to very poor.

*General health* included five questions to identify self-reported hypertension, high cholesterol, diabetes, arthritis, and joint or muscle pain. The questions were developed for this study and answered as dichotomous yes or no responses.

*Stress* was measured with the 10-item Perceived Stress Score Questionnaire (PSS-10) that assesses psychological stress and coping abilities [27,28]. The items were scored by reversing the scores on the four positive items (items 4, 5, 7, and 8) and summing the 5-point responses (never to very often) across all 10 items. Higher scores reflect greater stress and lower coping abilities. In principal component factor analysis, the positive and negatively worded items explained 48.9% of the total variance with an internal reliability of α = 0.78. Validity evidence showed inverse relations between the scale score and age, income, education, number of people living in a household and full-time employment in a professional occupation (p <.01). Construct validity showed significant correlations at p <.001 for stress measures related to life-events, job stress, workload demand, and self-reported stress [28].

*Physical activity* was measured with the self-administered, 7-item International Physical Activity Questionnaire (IPAQ) that assesses the frequency and duration of walking, moderate- and vigorous-intensity physical activity, and minutes spent sitting during past week. The scoring protocol from the IPAQ website ( http://www.ipaq.ki.se) was used to identify the MET-min^.^wk^-1^ of physical activity and to developed categories of low, moderate, and high physical activity. Evaluated in the 12-countries, the IPAQ MET-min^.^wk^-1^ score has acceptable one-week, test-retest reliability (r = 0.69-0.88); concurrent validity evidence against the long form of the IPAQ (r = 0.68 to 0.89); and criterion validity evidence against the CSA accelerometer’s total counts (r = 0.23) and time spent in moderate-vigorous intensity movement greater than 150 min^.^wk^-1^ (r = 0.74) [29].

*Dog walking* included three questions developed for this survey to identify the duration in hours and minutes of walking a dog on a typical day. A summary score was computed in minutes per day.

*Self-efficacy for exercise* was measured with a 5-item scale developed by Marcus et al. [30] to assess one’s confidence to continue exercising in different situations (e.g., when I am tired or when I am in a bad mood). The questionnaire was scored by summing the 7-point Likert responses (1 = not at all confident to 7 = very confident) with a higher score reflecting greater self-efficacy for exercise. This scale was shown to have acceptable two week test-retest reliability (0.90) and an internal consistency coefficient of 0.76 [30].

*Social support for exercise* was measured with the 5-item Physical Activity Social Support (PASS) developed by Eyler et al. [31]. The PASS instrument examines general support (1 question), friend (2 questions), and family support for exercise (2 questions) using a 4-point Likert response (1 = strongly agree to 4 = strongly disagree). The questionnaire was scored by dichotomizing responses to each question (0 = no support, 1 = support) and adding the scores together resulting in a score ranging from 0–5. The scale has been shown to have an internal consistency coefficient of 0.70 [31]. Furthermore, individuals reporting high levels of social support were more likely to be regularly active compared to individuals reporting low levels of social support and individuals with medium to high levels of social support were less likely to be sedentary [31].

*Neighborhood environment* was measured using an adaptation of four scales from a 26 item multi-dimensional survey developed by Mujahid et al. [32,33]. Specific scales used in the present study were walking (7 of 10 items), availability of healthy foods (3 of 4 items), safety (all 3 items), and social cohesion (all 4 items). All modules were scored by summing 5-point responses (1 = Strongly agree to 5 = strongly disagree) with lower scores reflecting better neighborhood conditions for physical activity and obtaining healthful foods. Validity tests of the original survey demonstrated internal consistency values of 0.73 for walking to 0.78 for availability of healthy foods. Test-retest correlations ranged from 0.62 for walking to 0.88 for safety [32].

*Mindfulness* was measured using the Mindfulness Attention Awareness Scale (MAAS) [34]. The 15-item instrument uses a 6-point Likert scale (almost always to almost never) and provides a global score of mindfulness that measures an individual’s tendency to pay attention to and to be aware of experiences in daily activities such as: how often the respondent encountered experiences such as acting on “automatic pilot’ or ‘being preoccupied’. The MAAS assesses individual differences in the frequency of states of mindfulness over time which can be indicative of decreased levels of perceived or psychological distress. Higher scores reflect higher levels of dispositional mindfulness. This survey has been shown to have an internal consistency coefficient of 0.82 in college students and 0.87 in adults [34]. The intraclass correlation for test re-test reliability over four weeks in college students was 0.81 [34].

#### Physical fitness and objective assessment of physical activity – sub-sample measures

*Accelerometry* was measured with the ActiGraph GT1M accelerometer (ActiGraph, Pensacola, FL) to provide an objective measure of physical activity movement during a one-week period in each measurement period. The ActiGraph provides information about the frequency, intensity, and duration of physical activity by utilizing a built-in single axis accelerometer which measures vertical accelerations at the hip. The accelerations are sampled at 30 times/sec, averaged over one minute, and outputted as numerical counts. The movement intensity is derived from the activity counts ranging from 0 to >10,000. The following cut-points were used to represent time spent at different intensities: inactivity or sedentary, 0–100 ct^.^min^-1^[35], light intensity, 101–759 ct^.^min^-1^, moderate lifestyle intensity, 760–5724 ct^.^min^-1^[36], moderate exercise/walking intensity, 1952–5724 ct^.^min^-1^, and vigorous intensity, ≥5725 ct^.^min^-1^[37]. The ActiGraph was worn for 7 days on a waist belt during all waking hours, except while in water. Participants were asked to record the time when the accelerometer was put on or removed at any time during the day. Minutes spent at movement intensities were summed across the days of wear time. Based on guidelines for using accelerometers in a field setting, a minimum of three days during the week with 10 h^.^d^-1^ of wear time was required for data to be included in the analysis [38].

*Anthropometric measures* were taken for height in centimeters (cm) using a portable stadiometer (Seca RoadRod 213). Height was measured at the top of the head in the horizontal plane. The participants were instructed to remove shoes and stand erect on the platform with their back against the vertical scale. Body weight was assessed in kilograms (kg) and percent body fat was estimated by bioelectrical impedance using a Tanita digital scale (Model TBF – 300 A, Arlington Heights, IL).Waist circumference was measured at the level of the umbilicus in duplicate to the nearest 0.5 cm with a Gulick II 150 cm tension controlled retractable tape [39]. If values were within 0.1 cm the mean of the two trials was recorded. If values were greater than 0.1 cm a third measure was taken. Only values within 0.5 cm of each other were used in analyses. Sagittal abdominal diameter was measured in duplicate to the nearest 0.1 cm at the level of the umbilicus with the subject lying supine with the knees extended [40] using a Holtain-Kahn abdominal caliper (Holtain, Ltd., Crosswell, Crymych; Dyfed, U.K). If values were within 0.1 cm the mean of the two trials was recorded. If values were greater than 0.1 cm a third trial was taken. Only values within 0.1 cm of each other were used in analyses. Body mass index was computed as weight in kg divided by height in meters squared.

*Resting blood pressure and resting heart rate* were measured using an Omron automated blood pressure cuff (HEM-711 DLX and HEM-7221-E, Shelton, CT). Blood pressure in mmHg and heart rate in b^.^min^-1^were taken after participants were seated for five minutes with an appropriately sized cuff. Two measures were made with a minimum of one minute between measurement trials. A third measure was taken if blood pressure values were greater than 4 mmHg difference. Only blood pressure values within 4 mmHg of each other and related heart rate values were used in analyses. The participants were asked to abstain from eating and smoking two hours before testing.

*Maximal oxygen uptake (VO*_*2*_*max)* was estimated using the Åstrand-Rhyming cycle ergometer test to examine cardiorespiratory fitness [41-43]. The Åstrand-Rhyming is a 6- minute, single work rate test completed using a model 839E Monark cycle ergometer (Monark Exercise, Vansbro, Sweden). Participants were asked to abstain from eating and smoking 2 hours before testing. Prior to testing participants were fitted with a heart rate monitor worn at the level of their heart, given instructions for the cycle test, and explained how to report their perceived exertion using Borg’s Rating of Perceived Exertion (RPE) scale [44]. The RPE uses a 6 – 20 point scale that describes exertion from very, very light to very, very hard. The cycle ergometer resistance was selected so participants would reach a steady-state heart rate of 120–150 beats per minutes (equivalent to 50-85% of their heart rate reserve computed as 220-age). The pedal rate was set at 50–60 revolutions per minute using a metronome set at 100 bpm.

The work rate was set at 50–75 Watts for untrained participants and up to 100 Watts for trained participants. If untrained participants could not maintain the assigned work rate, the work rate was lowered to 50 Watts or 25 Watts. Untrained participants were described as performing moderate intensity activity for less than 3 days a week and/or ≤30 minutes per day and trained participants were described as performing moderate-to-vigorous intensity activity 3 or more days per week and/or ≥30 minutes per day. The participants’ heart rate value was recorded continuously during the test using a telemetry system (Polar Electro Inc, Lake Success, NY). The test was initiated at the established work rate and continued for 6 minutes to increase the heart rate to a target range of 125 beats per minute to 85% of age-predicted heart rate max. If the heart rate was lower or higher than the target range, the workload was adjusted to bring the heart rate into the desired range and an additional 6 minutes of cycling was performed. The test was terminated when the difference in the heart rate between the 5th and 6^th^ minutes of exercise was 5 beats or less. If the difference in the heart rate values was greater than 5 beats, the test was continued until the heart rate between successive minutes was less than 5 beats or a maximum of 12 minutes of cycling was completed. The RPE was recorded during each minute of the tests. VO_2_ max (l^.^min^-1^) was estimated using the Åstrand-Rhyming nomogram from the steady state heart rate and the work rate. VO_2_ max adjusted for body mass (ml^.^kg^-1.^min^-1^) was computed as (VO_2_ max in l^.^min^-1^ × 1000)/kg body mass.

### Adherence

Participation in the study was determined from the participant’s recording of the steps taken each day. Failure to record steps for longer than one week was regarded as dropping out and accounted for the attrition observed during the study.

### Data collection and reduction

Step data were recorded every day onto the Select Wellness website. Average steps per week were computed across the 24 week study and steps for month 1, 3, and 6 were averaged using data from weeks 2, 3, 4, and 5 to reflect steps taken in the first month, weeks 11, 12, 13, and 14 to reflect steps taken in the third month and weeks 22, 23, 24, and 25 to reflect steps taken in the sixth month. Table 1 shows a diagram of the study outline and data collection schedules.

**Table 1:** The “ASUKI Steps study outline and data collection schedule

	**Recruitment**	**Pre-study**	**Intervention**						**Follow-up**
**Measure**	**Week**		**Month**						
	−4	−1	1	2	3	4	5	6	9
Pedometer steps			daily	daily	daily	daily	daily	daily	
Questionnaire^a^		x			x			x	x
Estimated VO_2_ max (ml^-1.^kg^-1.^min^-1^)^b^			x					x	x
Weight (kg)			x		x			x	x
Height (cm)			x		x			x	x
Blood Pressure (mmHg)			x		x			x	x
Waist circumference (cm)			x		x			x	x
Sagittal diameter (cm)			x		x			x	x
Body fat percent			x		x			x	x
ActiGraph accelerometer			x		x			x	x

^a^ Questionnaire items included demographic data, health status, quality of life, readiness for exercise, self-efficacy for exercise, social support for exercise, stress and anxiety, dog walking, physical activity, sleep, mindfulness, neighborhood supports for physical activity and diet.

^b^*Abbreviations*: VO_2_ max ml^-1.^kg^-1.^min^-1^ = maximal oxygen uptake in milliliters per kilogram body weight per minute; kg = kilograms; cm = centimeters; mmHG = millimeters of mercury.

### Statistical analysis

Questionnaire data and steps per day were recorded in an excel database. Physical fitness data were recorded onto forms used for direct data entry with the Cardiff TeleForm system (Vista, CA) and stored in an excel database. Accelerometer data were downloaded using proprietary software (ActiGraph Inc.) to extract the movement counts. Minutes spent at different intensities were computed using a SAS 9.2 (Cary, NC) program for this purpose. The study data will be examined for outliers and normality. Mixed models ANOVA will be used to assess the fixed and random effects of the intervention on the study aims. Linear and polynomial regression growth model analyses will be used to assess the rate of change in study variables over the six month study period.

### Limitations

Due to logistical reasons of enrolling nearly 2,000 participants from two universities, each with two to three campuses, and because just giving a pedometer to people and asking them to report their physical activity is an intervention in itself, we were unable to collect pedometer data before the start of the study. Hence, “ASUKI Step” does not have a true baseline of the usual steps per day walked before the study began. However, the study is able to show individual changes in steps taken during the study and concomitant changes in correlates of walking behaviors during the 6 month study. Baseline data are available for laboratory assessments with accelerometry, body composition, and cardiorespiratory fitness. The questionnaire data were collected prior to the participants beginning the walking intervention.

## Discussion

“ASUKI Step” is a quasi-experimental, worksite pedometer-based physical activity intervention designed to increase the number of days employees walk 10,000 steps and to reduce the number of days employees spend being inactive, defined as taking less than 3,000 steps per day. The study also describes the intra- and interpersonal determinants for success in the intervention and in a sub-sample of the study, changes in physical fitness and body composition during the study.

## Competing interests

The authors have no competing interests, financial or non-financial, to declare.

## Authors’ contributions

BEA participated in the design of the study, collected data, prepared the data set, and drafted the manuscript. CD participated in the design of the study, analyzed data, and revised the manuscript critically for important intellectual content. AS participated in subject recruitment, data collection, data analysis, and revised the manuscript critically for important intellectual content. JW participated in subject recruitment, data collection, data analysis, and revised the manuscript critically for important intellectual content. PS participated in the design of the study, interpretation of the data, and reviewed the manuscript critically for important intellectual content. EP participated in subject recruitment, data collection, data analysis, and reviewed the manuscript critically for important intellectual content. AY conceived the study, participated in the design of the study, data collection and analysis, and helped to draft the manuscript. All authors read and approved the final manuscript.

## Pre-publication history

The pre-publication history for this paper can be accessed here:

http://www.biomedcentral.com/1471-2458/12/657/prepub
